# Characteristics of successful changes in health care organizations: an interview study with physicians, registered nurses and assistant nurses

**DOI:** 10.1186/s12913-020-4999-8

**Published:** 2020-02-27

**Authors:** Per Nilsen, Ida Seing, Carin Ericsson, Sarah A. Birken, Kristina Schildmeijer

**Affiliations:** 1 Department of Medical and Health Sciences, Division of Community Medicine, University of Linköping, Linköping, Sweden; 2 Department of Behavioural Sciences and Learning, University of Linköping, Linköping, Sweden; 3 Cardiology and Speciality Medicine Centre, Region Östergötland, Linköping, Sweden; 4 Department of Health Policy and Management, Gillings School of Global Public Health, The University of North Carolina at Chapel Hill, Chapel Hill, North Carolina USA; 5 Department of Health and Care Sciences, Linnaeus University, Kalmar, Sweden

**Keywords:** Organizational change, Implementation, Influence, Preparedness, Patient benefit

## Abstract

**Background:**

Health care organizations are constantly changing as a result of technological advancements, ageing populations, changing disease patterns, new discoveries for the treatment of diseases and political reforms and policy initiatives. Changes can be challenging because they contradict humans’ basic need for a stable environment. The present study poses the question: what characterizes successful organizational changes in health care? The aim was to investigate the characteristics of changes of relevance for the work of health care professionals that they deemed successful.

**Methods:**

The study was based on semi-structured interviews with 30 health care professionals: 11 physicians, 12 registered nurses and seven assistant nurses employed in the Swedish health care system. An inductive approach was applied using questions based on the existing literature on organizational change and change responses. The questions concerned the interviewees’ experiences and perceptions of any changes that they considered to have affected their work, regardless of whether these changes were “objectively” large or small changes. The interviewees’ responses were analysed using directed content analysis.

**Results:**

The analysis yielded three categories concerning characteristics of successful changes: having the opportunity to influence the change; being prepared for the change; valuing the change. The interviewees emphasized the importance of having the opportunity to influence the organizational changes that are implemented. Changes that were initiated by the professionals themselves were considered the easiest and were rarely resisted. Changes that were clearly communicated to allow for preparation increased the chances for success. The interviewees did not support organizational changes that were perceived to be implemented unexpectedly and/or without prior communication. They conveyed that it was important for them to understand the need for and benefits of organizational changes. They particularly valued and perceived as successful organizational changes with a patient focus, with clear benefits to patients.

**Conclusions:**

Organizational changes in health care are more likely to succeed when health care professionals have the opportunity to influence the change, feel prepared for the change and recognize the value of the change, including perceiving the benefit of the change for patients.

## Background

The only constant in health care organizations, as the saying goes, is change. Technological advancements, ageing populations, changing disease patterns and new discoveries for the treatment of diseases require health care organizations and professionals to change almost constantly [1–4]. Organizational changes are also needed to account for evolving societal norms and values, some of which have yielded higher expectations for access to health care, improved patient experience and increased patient involvement in care decision making [5, 6]. Continuous professional education has become increasingly important to ensure that health care professionals’ competencies keep pace with current standards and to maintain and enhance the knowledge and skills needed to stay abreast of the newest evidence [7].

Organizational changes affecting health care professionals also relate to political reforms and policy initiatives. The advent of New Public Management (NPM) has challenged the traditional professional dominance, introducing a logic of managerialism into health care, i.e. work should be organized and controlled by managers to achieve organizational goals of a cost-effective and efficient health care [8]. Health care professionals are increasingly expected to document their work, take on administrative tasks and participate in management-led quality improvement initiatives [9]. Changes also relate to the evidence-based movement, which has emerged in the wake of NPM, with ambitions to provide a stronger scientific foundation for professional practice [7].

In general, changes can be challenging because they contradict humans’ basic need for a stable environment [10, 11]. Research has shown that organizational changes are often associated with employees’ psychological uncertainty about how the changes will affect their work situation, role and overall life [3, 12, 13]. High rates of organizational change have well-documented effects on employee health and well-being, as assessed by a range of indicators, e.g. reduced organizational commitment, loss of productivity, work-related stress, emotional exhaustion, mental health problems, change fatigue, poor self-rated health, adverse sleep patterns, sickness absence, hospital admissions and stress-related prescriptions [14–16].

Many changes in organizations fail to achieve desired goals; a 70% failure rate is commonly cited [17]. While generic success or failure rates can be questioned due to the context-dependent nature of change and challenges regarding definitions and measurement, there is still a considerable proportion of changes that do not fail. This is the premise for the present paper: what characterizes successful organizational changes in health care? Based on interviews with health care professionals in Sweden, we aimed to investigate the characteristics of changes of relevance for the work of health care professionals that they deemed successful. Knowledge of conditions associated with successful organizational change has the potential to improve selection, planning, implementation and management of ubiquitous changes in health care organizations.

## Methods

### Study setting, design and participants

Study data come from interviews with Swedish health care professionals (physicians, registered nurses, assistant nurses). In the Swedish health care system, residents are insured by the government, with equal access to health care for the entire population, although private health care also exists. Sweden’s 21 regions are responsible for providing health care.

We conducted semi-structured individual interviews with 11 physicians, 12 registered nurses and seven assistant nurses – 30 health care professionals total (Table 1). The health care professionals were employed in six different health care units located in small- to mid-sized cities in south-eastern Sweden (populations of 67,000, 135,000 and 150,000 inhabitants, respectively).

**Table 1:** Participant characteristics

Characteristics	Physicians (*n* = 11)	Registered nurses (*n* = 12)	Assistant nurses (*n* = 7)
Gender, n (%)			
Male	5 (45.5)	1 (8.3)	1 (14.3)
Female	6 (54.5)	11 (91.7)	6 (85.7)
Years in the occupation, n (%)			
0–9 years	0 (0)	1 (8.3)	0 (0)
10–20 years	7 (63.6)	3 (25)	0 (0)
21 or more years	4 (36.4)	8 (66.7)	7 (100)
Median years in the occupation	17	28	30
Years in the current workplace, n (%)			
0–9 years	4 (36.4)	6 (50)	4 (57.1)
10–20 years	5 (45.4)	3 (25)	3 (42.9)
21 or more years	2 (18.2)	3 (25)	0 (0)
Median years in the current workplace	17	9	9
Workplace, n (%)			
Internal medicine, endocrine and cardiology	3 (27.3)	4 (33.3)	2 (16.7)
Surgery clinic	1 (9.0)	1 (8.3)	1 (8.3)
Orthopaedic clinic	2 (18.2)	1 (8.3)	1 (8.3)
Emergency care clinic	2 (18.2)	1 (8.3)	1 (8.3)
Primary health care unit	3 (27.3)	3 (25.0)	2 (16.7)
Other	0 (0)	2 (16.7)	0 (0)

To achieve a sample of health care professionals that represented a broad spectrum of perceptions and experiences concerning changes in health care – i.e., working in primary, secondary and tertiary health care facilities serving patients who varied in terms of health status and duration of stay – we used a purposeful sampling strategy.

To recruit frontline health care professionals, we used an e-mail that briefly described the study. We sent the e-mail request to the manager of each work unit, with a request that they forward our request to physicians, registered nurses and assistant nurses. We then sent an informational letter describing the study to those who responded to our email. No one declined to participate after receiving the information letter. We scheduled interviews at a time (between January and September 2018) and in a location convenient to participants, where they could feel comfortable about speaking honestly (e.g. office with a closed door).

### Data collection

We used an inductive approach to data collection, with a semi-structured interview guide developed by the authors. The interview guide is available as an Additional file. Interview questions were based on the existing literature on organizational change and change responses [15, 18–20] and concerned the participants’ experiences and perceptions of any changes that they considered to have affected their work. Of note, we asked participants to consider changes ranging from “objectively” large organizational changes, e.g. a re-structuring of the organization, to small changes, e.g. modification of an already existing workplace routine. This approach allowed us to assess both broad, more general changes as well as more specific examples of changes, such as the merging of the informant’s work unit with another unit, introduction of new information technology systems, or moving to new localities.

Although individuals’ subjective experience may not correspond with more objective measures of organizational outcomes of changes, it is crucial to understand health care professionals’ views on changes in health care because their attitudes towards changes may influence changes’ success [21, 22]. As such, instead of asking about specific changes or providing lists or examples of changes, we allowed the participants to discuss any changes they considered to be relevant to their work; this approach reflects research that shows that experiences of are often individual (e.g., one change may be attractive and imply advantages for some and be a source of stress and disadvantages for others) [23].

We began each interview with questions about the participant, the content of their work, and their workplace. We then asked participants to describe examples of organizational changes that they considered to be successful. Then, we asked participants to offer a rationale for these changes’ success. We asked a final open-ended question to capture any other reflections that participants had.

In two interviews, we pilot tested the questions to assess their meaningfulness and clarity of concepts. Pilot interview results suggested that the questions could be used in different health care contexts, that the wording was clear, and that the interview fit within participants’ maximum available time (60 min). We included the two pilot interviews in the study.

Individual interviews were conducted by all the authors except SB, who does not speak Swedish, and were digitally recorded. Before the start of an interview, the participant was asked to re-read the information letter and give written informed consent to participate. Each interview lasted between 28 and 104 min (mean, 50.5 min). The interviews were transcribed verbatim by a professional transcription agency and were then reviewed by the researcher who conducted the interview.

### Data analysis

Using an inductive approach, participants’ responses were analysed using directed content analysis according to descriptions by Hsieh and Shannon [24]. All authors except SB read the transcripts of the interviews individually to create a holistic view of the material. In the next step, each researcher performed a first analysis condensing meaning-bearing units and creating codes and subcategories. PN, IS, CE and KS then met to discuss and compare their respective interpretations of the material. Tentative findings were reported to and discussed with SB. Following her input, PN, IS, CE and KS met again (as SB is located in the US) to discuss the preliminary findings. This discussion led to a proposal concerning the categories of analysis, which was then fed back to SB for her comments. Eventually, consensus was reached on the categories and PN suggested labels which were accepted by the whole group. Representative quotations for reporting were jointly identified by PN, IS, CE and KS. PN, who is fluent in English, then translated the quotations from Swedish to English, which were then examined by IS, CE and KS for accuracy. Finally, SB, whose first language is English, reviewed the English-language quotations for clarity.

## Results

The analysis yielded three categories concerning characteristics of successful changes: having the opportunity to influence the change; being prepared for the change; valuing the change. The findings regarding these characteristics were equally applicable to the physicians, registered nurses and assistant nurses, with few notable differences among the three professional categories. The quotes are attributed to the physicians (P), registered nurses (RN) and assistant nurses (AN), who were interviewed, numbered from 1 to 30.

### Having the opportunity to influence the change

The health care professionals emphasized the importance of having the opportunity to influence organizational changes that are implemented. Changes that were initiated by the professionals themselves were considered the easiest and rarely encountered resistance on the part of health care professionals. A physician (4P) described the importance of “bottom-up” changes, “I think one is particularly responsive to issues that are being raised in the organization from the ground up. It is from there, I think, most often the smartest ideas will emerge, but then it is important to ensure that you are responsive and assess [the ideas].” An assistant nurse (1AN) expressed a similar view, “It’s a good change, I believe, [if] it’s a change that has occurred with me being involved from the start and built [from there].” The health care professionals suggested that they are most knowledgeable about their work, putting them in an optimal position to identify relevant problems and initiate appropriate changes.

Concerning organizational changes initiated by the health care management and/or the higher political leadership level in the region, the health care professionals suggested that being involved early in the change process and being able to have an influence throughout the change process contributed to the change’s success. For example, a registered nurse (2RN) said, “If employees are involved from the beginning and believe this [change] is interesting, then I think there is a chance to succeed [with the change].” However, many complained about the difficulty of influencing changes because of the hierarchy of the health care system and the long distance to those in power over most changes. A physician (13P) opinioned, “We don’t have any channels to the political level or other higher management levels. You’re restricted to the head of the clinic to be your spokesperson.” Another physician (23P) complained, “There are administrators or controllers or economists who look into the [health care] system, but they lack knowledge about the actual care work, which makes me angry. They start their project, but don’t involve us.”

### Being prepared for the change

According to the health care professionals, organizational changes that were clearly communicated to allow for preparation increased the chances for successful changes. An assistant nurse (22AN) argued that a relatively slow tempo of change is important when implementing change, “It [i.e. the change] has to proceed at a calm pace so that everyone is part of it, so that you have a shared plan, that’s the most important thing, I think.” A registered nurse (5RN) talked about the importance of how changes are communicated, “I can’t take it all in, I can’t handle it. You get this flow of mails with information, ‘Now we will do this and that, now this will change and this is the starting date…’ It can be from day to day, we cannot catch up.”

The health care professionals did not support organizational changes that were perceived to be implemented unexpectedly and/or without prior communication. One of the physicians (3P) described such a change: “Discussions were ongoing during the autumn, but you felt that the management didn’t listen. Then came January with the decision: ‘You will be split, in two weeks you will be two different clinics.’ We felt so powerless and uninformed. We had two weeks to develop new systems and that results in considerable consequences.” A registered nurse (21RN) also lamented a lack of time for preparation, “We had quick meetings. Sure, we met and talked about it [the change], but we didn’t have much time. We had to solve it anyway.”

### Valuing the change

The health care professionals conveyed that it was important for them to understand the need of organizational changes and how they benefitted themselves and/or the patients. The changes might otherwise be perceived as meaningless and unjustified, which may create change resistance. A physician (24P) stated, “I want to see a purpose for it [i.e. the change], and if I do [recognize the value of the change], and it works, then I’m satisfied.” Similarly, a registered nurse (5RN) emphasized the importance of the health care professionals recognizing the value of the change, “We need to feel that this change is not done because the region has decided it, but because we really believe that it will make things better.”

In particular, health care professionals valued and perceived as successful organizational changes with a patient focus, with clear benefits to patients. According to a registered nurse (12RN), “As long as you see that it [i.e. the change] benefits our patients, I think you have quite considerable motivation.” Further, an assistant nurse (22AN) said that “one does it to make it easier for the patients and maybe for the staff, that’s the most important.”

## Discussion

Change is pervasive in modern health care. This study aimed to identify characteristics of successful organizational changes from the perspective of health care professionals at the frontline level of health care. An important premise for the study was that the health care professionals’ subjective experiences of changes influence the likelihood of achieving successful changes. The importance of individual responses to organizational changes has been increasingly emphasized [25].

Three categories (i.e. characteristics of successful changes) were found to be of central importance for a change to be considered successful according to the statements of the health care professionals who were interviewed: that health care professionals (1) have the opportunity to influence the change, (2) are prepared for the change and (3) recognize the value of the change. Many of the statements by the participants were representative of more than one category, suggesting an interdependency between the three categories of this triad of successful change characteristics. For example, a slower change allows for preparation, which facilitates involvement and influence, thus enabling an appreciation for the change. Alternatively, recognizing the value of a change, e.g. its patient benefits, likely contributes to increased motivation among health care professionals to become engaged and participate in carrying out the change. This interdependence implies that successful change is more likely if more than one of the three categories is accounted for when planning and implementing changes. The importance of preparation for and involvement in a change has been associated with decisional latitude [26] and valuing the change in terms of experiencing personal gains has been linked with involvement in the change [27]. However, we have not been able to find any previous study, either in health care settings or in other environments, which has identified the relevance of this particular triad of characteristics or how they are interlinked. Although our findings suggest these interdependencies, we did not collect data to specifically investigate the underlying mechanisms; thus, exploring these interdependencies would be an important area for future research.

The health care professionals in our study attached great importance to being able to influence changes that may influence their work. They expressed positive attitudes to changes that have been developed and emanate “bottom up” from themselves and/or the frontlines of health care. Many of the health care professionals complained about the power differential between those who are affected by the changes and higher management and political levels of the health care system who usually decide on what changes to implement. Physicians in Sweden have often raised complaints that policy making and decisions concerning the medical profession are made without physicians or their professional organizations being involved in the decision-making process [28]. These findings underscore the importance of changes having frontline support and being perceived as legitimate among the employees affected by the changes.

Organizational research has shown that participation in changes can yield increased acceptance. Indeed, widespread participation in the change process is perhaps the most frequently cited approach to overcoming resistance to change [29, 30]. Even assuming a well-justified and well-planned change initiative, research underscores the importance of managers building internal support for change by means of employee participation in the change process [31]. These are common findings in organizational research in general, but they seem particularly applicable in health care organizations because of the strong professional discretion in performing the work.

Health care professionals emphasized the importance of predictability for them to perceive organizational changes as successful. Individuals are better able to adjust their behaviour accordingly when they are prepared [3]. The importance of managers’ communication of information to prepare employees for organizational changes is often pointed out in the organizational change literature [31]. However, despite the relevance of predictability, many changes in our study seemed to be characterized by a lack of preparation. When individuals are unprepared, they have difficulties aligning their thoughts, feelings and behaviours with the expectations of those who lead the changes [12, 32]. Our findings are consistent with Organizational Readiness to Change, a theory that posits that readiness depends on organization members’ resolve to pursue the courses of action involved in implementing change (change commitment) and their beliefs in their capabilities to execute these actions (change efficacy). Contextual factors such as resources and culture also influence their preparedness to implement change [33].

The importance of management communicating the motives for changes was stressed by the health care professionals in our study. Consistent with our findings, organizational change research has demonstrated that changes have a greater chance of succeeding if employees consider them to be well thought out and respect the managers responsible for the changes, whereas resistance to changes is more likely if employees consider the changes to have little or no value for themselves [31]. The organizational change literature also stresses the importance of change initiatives resting on coherent and sound causal thinking [34–36]. Employees who do not understand why a change is pursued will be reluctant to comply with the management’s requirement for the change [25]. The health care professionals in our study argued that the changes must benefit patients to have value. This is consistent with research that shows that health care professionals’ role identity is largely defined by patients and patients’ needs [37].

The overall findings of our study may reflect a tension between the traditional logic of professionalism and the managerial logic introduced into health care with the emergence of NPM. Whereas the logic of managerialism assumes that work should be management led to achieve organizational goals, health care professionals tend to be loyal to their profession and their emotional rewards at work are primarily associated with their patients [9]. NPM has led to an increase in the use of management systems, e.g. auditing, guidelines, recommendations, adverse event reporting systems and various incentive tools [38] that challenge the logic of professionalism in terms of professionals’ autonomy and freedom of judgement in performing their work [39–41]. According to professional theory, true professionals such as physicians and lawyers independently treat individual cases (e.g. patients and clients) and make decisions based on their knowledge and skills; they are highly educated and trained to apply knowledge and expertise in solving complex problems [42, 43]. Research suggests that physicians due to their stronger identification with professional logic are more likely than nurses to be critical of management-initiated changes [9]. Several studies have shown how physicians respond with scepticism or suspicion to different forms of management-led changes in health care [44, 45].

Sweden has seen a lively public debate on NPM in recent years, with many scholars, policy makers and both physicians and registered nurses critiquing core NPM principles and their consequences for health care professionals [46–50]. In response to the criticism of NPM principles, the Swedish government has recently introduced the concept of “trust-based governance,” intended to integrate aspects of professional logic with NPM-based managerial logic, thus providing an alternative to governing health care professionals through auditing, control and performance management [26, 51]. Governance by trust is intended to let “the professionals be professional” [52]. This initiative is new and we are not aware of any studies of the concept, but research is warranted to investigate how this concept is realized in practice. Future research should assess whether health care professionals perceive changes as more successful under trust-based governance than under NPM principles.

The results of our study should be evaluated in the context of the methods that we chose to address our study question. We chose a qualitative approach because little is known about responses to changes in Swedish health care. For this reason, we considered interviews with physicians, registered nurses, and assistant nurses to gain a deeper understanding of the topic. Participation was voluntary; the interviewees were selected and asked by their respective supervisors about participation in the study, which means that the participants may have been particularly interested in the subject.

The multidisciplinary research team was a strength of the study, because it allowed different perspectives on the issue of changes in health care. The team consisted of the following professions: behavioural economist (PN), political scientist (IS), registered nurse (KS), behavioural scientist (CE) and organizational sociologist (SB). Another strength was the relatively high number of interviews (*n* = 30), although Malterud et al. [53] emphasizes that the strength of the information received (information power) is more important than the size of the sample. Regardless, this enabled us to use quotations from many different participants, adding transparency and trustworthiness to the findings.

The main contribution of the study lies in identifying a “triad of successful change characteristics” from the change recipients’ point of view. While many findings of the study are in line with existing research on organizational changes, no previous study has identified this particular triad of interdependent characteristics. The study provides important knowledge for health care organizations to plan and implement changes with better chances of being successful.

## Conclusions

In conclusion, organizational changes in health care are more likely to succeed when health care professionals have the opportunity to influence the change, feel prepared for the change and recognize the value of the change, including perceiving the benefit of the change for patients. Although changes in health care organizations are inevitable, there are more or less effective ways to carry out changes. Our results provide important implications for health care organizations concerning how changes in health care can be planned, implemented and managed to increase the chances that they will be supported by health care professionals, which is crucial for successful changes.

## Supplementary information

**Additional file 1.** Interview guide.

## Data Availability

All interview data analysed during the current study are available from the corresponding author on reasonable request.

## Abbreviations

AN: Assistant nurses
CE: Carin Ericsson
IS: Ida Seing
KS: Kristina Schildmeijer
NPM: New Public Management
P: Physicians
PN: Per Nilsen
RN: Registered nurses
SB: Sarah A. Birken
US: United States
