# Supplementary material for: Characteristics of successful changes in health care organizations: an interview study with physicians, registered nurses and assistant nurses
Source: BMC Health Serv Res. 2020 Feb 27;20:147. doi: 10.1186/s12913-020-4999-8 (PMC7045403; doi:10.1186/s12913-020-4999-8)
Supplement: Supplementary file 1 — Additional file 1. Interview guide. [file 12913_2020_4999_MOESM1_ESM.docx]

INTERVIEW GUIDE

Introduction

Note gender, age, profession and years in the profession

[Person]

1 Can you tell me about yourself: education, employee for how long at this workplace, different employment in the Region or other work experience?

[Job]

2 Can you tell us a little about your workplace and how it is organized, e.g. number of employees, gender distribution, managers, patient categories?

*[Read:] This project is about changes that occur in healthcare, and how they affect physicians, nurses and patients. By changes we mean everything from small changes (e.g. a new work routine) to large changes (e.g. a major re-organization). Examples may be changes in documentation, working methods, digitization/IT, treatment methods and medicine – anything that affects your work.*

[Inventory / classification of changes]

3 Have there been any changes in your workplace during the time you have worked here? [If so:] What kind of changes have occurred? Can you give examples of major changes that have taken place?

Probes:

• Have there been, or are there, many changes?

• To what extent have the changes increased or decreased over time?

• Has the nature of the changes changed during the time you have worked here? [If yes:] How?

[Personal experiences / experiences of change]

4 Please tell me about your experiences of changes in your workplace. It may be changes that have taken place in the past, in the present or planned changes.

[Successful and failed changes]

5A Can you describe examples of changes in your workplace where the intended result was achieved? If so, why were they successful, in your opinion?

5B Can you describe examples of changes in your workplace where the intended result failed? If so, why did they fail, in your opinion?

*[Read:] In the project we investigate if and how changes in healthcare lead to feelings of stress and change fatigue (CF).*

[When does change create CF?]

6 What changes can create - according to your own experience or what you have seen among colleagues - negative feelings? And what changes do not contribute to such fatigue?

[Own experience / experience of FT]

7 Can you describe what feelings arise regarding changes you experience as negative?

[Consequences # 1]

8 How does CF affect health and well-being that you have experienced yourself or seen among colleagues?

[Consequences # 2]

9 How does CF affect the work situation, job satisfaction and work environment that you have experienced yourself or seen among colleagues?

[Consequences # 3]

10 Do you believe that the quality of care is affected by staff CF? If so, how?

[Experience of strategies]

11 Do you have any experience of employers / supervisors / managers having strategies for reducing CF? If so, what measures has the strategy included? How did these turn out?

[Own suggestions on strategies]

12 Do you have any suggestions for possible strategies to prevent CF?

[Termination]

13 Is there anything else you want to add before we finish the interview? Is there anything I haven’t asked about that you think I should have included?
